# The Evolving Clinical Management of Genitourinary Cancers Amid the COVID-19 Pandemic

**DOI:** 10.3389/fonc.2021.734963

**Published:** 2021-09-27

**Authors:** Sudeh Izadmehr, Dara J. Lundon, Nihal Mohamed, Andrew Katims, Vaibhav Patel, Benjamin Eilender, Reza Mehrazin, Ketan K. Badani, John P. Sfakianos, Che-Kai Tsao, Peter Wiklund, William K. Oh, Carlos Cordon-Cardo, Ashutosh K. Tewari, Matthew D. Galsky, Natasha Kyprianou

**Affiliations:** ^1^ Department of Medicine, Division of Hematology/Medical Oncology, Icahn School of Medicine at Mount Sinai, New York, NY, United States; ^2^ Department of Pathology, Molecular and Cell-Based Medicine, Icahn School of Medicine at Mount Sinai, New York, NY, United States; ^3^ Tisch Cancer Institute, Icahn School of Medicine at Mount Sinai, New York, NY, United States; ^4^ Department of Urology, Icahn School of Medicine at Mount Sinai, New York, NY, United States; ^5^ Department of Oncological Sciences, Icahn School of Medicine at Mount Sinai, New York, NY, United States

**Keywords:** SARS-CoV-2, COVID-19, penile cancer, prostate cancer, testicular cancer, renal cancer, bladder cancer

## Abstract

Coronavirus disease–2019 (COVID-19), a disease caused by Severe Acute Respiratory Syndrome Coronavirus 2 (SARS-CoV-2) infection, has become an unprecedented global health emergency, with fatal outcomes among adults of all ages throughout the world. There is a high incidence of infection and mortality among cancer patients with evidence to support that patients diagnosed with cancer and SARS-CoV-2 have an increased likelihood of a poor outcome. Clinically relevant changes imposed as a result of the pandemic, are either primary, due to changes in timing or therapeutic modality; or secondary, due to altered cooperative effects on disease progression or therapeutic outcomes. However, studies on the clinical management of patients with genitourinary cancers during the COVID-19 pandemic are limited and do little to differentiate primary or secondary impacts of COVID-19. Here, we provide a review of the epidemiology and biological consequences of SARS-CoV-2 infection in GU cancer patients as well as the impact of COVID-19 on the diagnosis and management of these patients, and the use and development of novel and innovative diagnostic tests, therapies, and technology. This article also discusses the biomedical advances to control the virus and evolving challenges in the management of prostate, bladder, kidney, testicular, and penile cancers at all stages of the patient journey during the first year of the COVID-19 pandemic.

## Introduction

The novel severe acute respiratory syndrome coronavirus (SARS-CoV-2) is a positive sense single-stranded enveloped RNA virus which belongs to the Coronaviridae family of viruses, that has rapidly spread since being identified in Wuhan, China in December 2019 (1), with 202 million confirmed current cases worldwide (2). The introduction of the novel virus has changed the practice of healthcare universally. The consequences of these changes, in order to treat, curb, and mitigate the spread of the virus, are being explored, especially for cancer patients. Medical care for cancer patients was halted due to patient fear, stay at home orders, social distancing, and the shift in medical practice to care for those impacted by SARS-CoV-2.

In this review, we present a timely overview of the medical and scientific findings to date on the impact of SARS-CoV-2 on genitourinary (GU) cancers, including prostate, bladder, kidney, testicular, and penile cancers, over the past year, as we mark the first year of the coronavirus disease 2019 (COVID-19) pandemic, and offer recommendations for the diagnosis, clinical care, and therapeutic management of GU cancer patients during the ongoing pandemic. Furthermore, we discuss the potential synergy between COVID-19 and cancer leading to adverse clinical outcomes in SARS-CoV-2 infected cancer patients, and the advancement in innovative technology usage amid the COVID-19 pandemic in GU oncology practice.

## SARS-CoV-2 Mechanism of Infection

SARS-CoV-2 is a highly contagious positive-sense single-stranded RNA virus that was first described as a cluster of severe pneumonia cases in Wuhan, China on December 31, 2019, and subsequently identified as a coronavirus on January 7, 2020 by the Chinese Center for Disease Control and Prevention (1). The virus causes COVID-19. The World Health Organization (WHO) declared COVID-19 a global pandemic on March 11, 2020 (1). As of August 9 2021, the WHO has reported over 202.7 million global cases with over 4.3 million deaths worldwide, of which 78.7 million cases were in the Americas, 61.3 million in Europe, 39.3 million in South Asia, 13.2 million in Eastern Mediterranean, 5.2 million in Africa, and 5.0 million in the Western Pacific (2). SARS-CoV-2 is transmitted between humans by respiratory droplets with common symptoms such as fever, sore throat, dry cough, shortness of breath, and fatigue, with some patients developing severe COVID-19 and requiring hospitalization (3). Mortality from COVID-19 is higher in men, individuals over the age of 65, and patients with comorbidities (4). The genomic sequence of SARS-CoV-2, isolated from a patient in Wuhan, China, was provided on January 10, 2020 to support worldwide research and global diagnostic and therapeutic efforts (1, 5).

SARS-CoV-2 uses two host cell proteins to enter and replicate within a cell: angiotensin-converting enzyme 2 (ACE2) and the cell surface transmembrane protease serine 2 (TMPRSS2). Researchers have turned to single cell RNA-sequencing to identify cell types that co-express ACE2 and TMPRSS2 (6, 7). Interestingly, findings by Singh et al. identified several genitourinary organs (prostate, kidney, and testis) that co-express ACE2 and TMPRSS2 and may serve as a viral reservoir and site of infection (8). Emerging clinical and molecular biology data from COVID-19 patients established that SARS-CoV-2 is primarily detected in broncho-alveolar lavage fluid, sputum, nasal swabs, less commonly in fibro-bronchoscope brush biopsies, pharyngeal swabs, and feces, and to an even lesser extent in blood and urine (8, 9). The gender disparity relating to infectivity and severity of COVID-19, in which males have a higher rate of hospitalization and mortality than females, may reflect the importance of the prostate and testis as viral reservoirs and/or the impact of androgens on COVID-19 (4). Currently, over 4.46 billion vaccine doses have been administered worldwide in an effort to curb viral transmission.

## Advocating for GU Cancer Diagnosis During COVID-19

**Figure 1:**
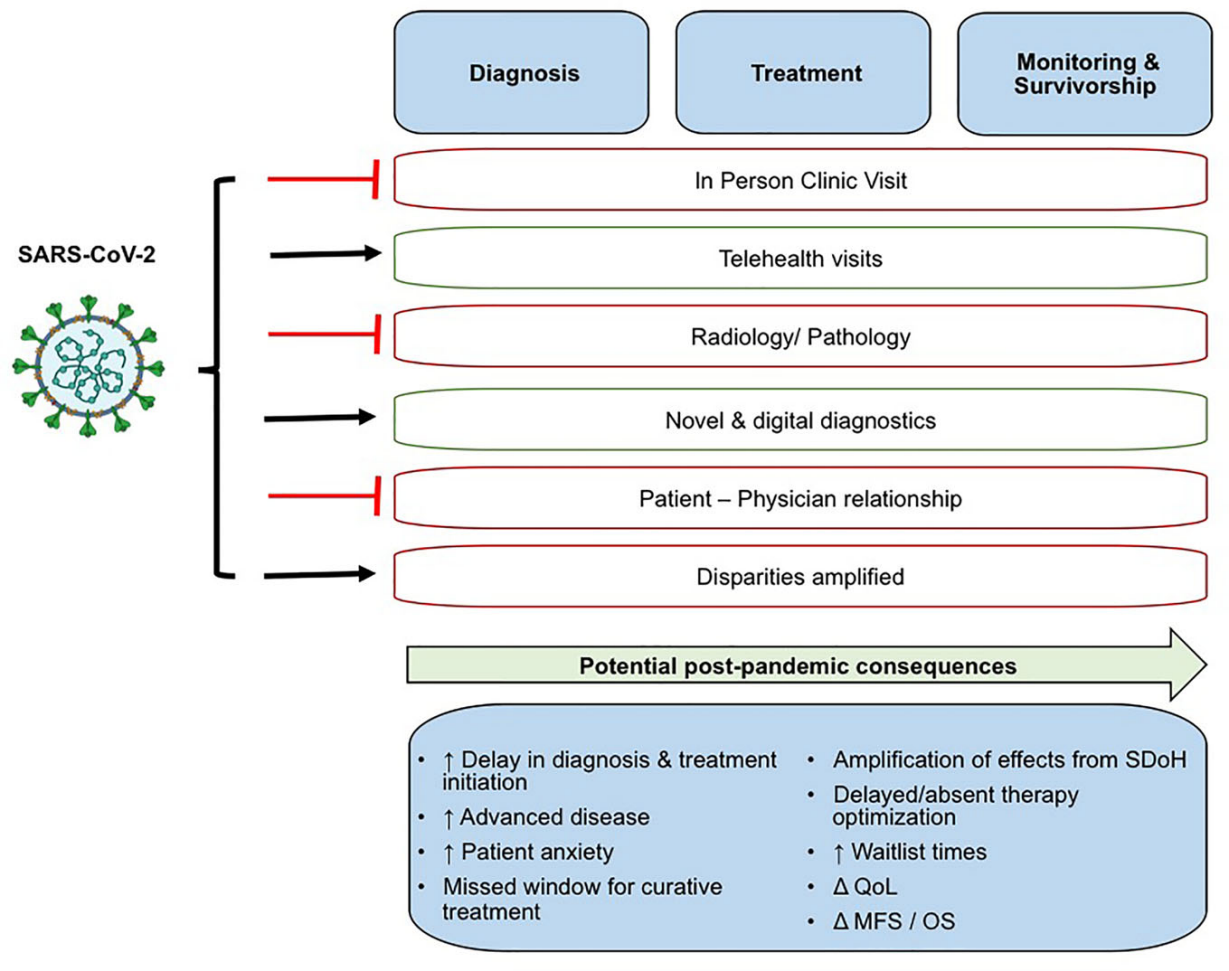
The effects of COVID-19 on the diagnosis, treatment, management, survivorship and disease monitoring in patients with GU cancers. QoL, Quality of Life; MFS, metastasis free survival; OS, overall survival.

The effects of COVID-19 on the diagnosis, treatment, management, survivorship and disease monitoring in patients with GU cancers are summarized in **Figure 1**. COVID-19 disproportionately affects older males, both risk factors shared with many GU cancers. Bladder, prostate, penile, and kidney cancer patients, on average, are over 60 years of age and fall into the high-risk age group if exposed to the virus. SARS-CoV-2 infected men are disproportionately affected by COVID-19 compared to women, with respect to mortality and morbidity (10). Physicians have been weighing the risks of prolonging cancer diagnosis and postponing treatment against the dangers of contracting SARS-CoV-2 for the cancer population (11). Due to the prevalence and contagious nature of SARS-CoV-2, triage of GU cancer patients is necessary given their immunocompromised state and increased risk of developing severe complications from COVID-19, and varying risk of disease progression (11–14). The rapid demonstration of a biological and clinical interaction between SARS-CoV-2 infection and cancer pathobiology, suggests that cancer patients are more likely to become infected by SARS-CoV-2, highly likely to develop severe COVID-19 infection, and to die from COVID-19 (15). COVID-19 and cancer act synergistically, and cancer patients with co-morbidities have worse outcomes (16, 17). However the shared mechanisms of COVID-19 and different cancer subtypes may vary.

Early evidence from the CCC-19 registry study in the USA suggests that surgery in cancer patients does not increase COVID-19 mortality; of note 16% of this cohort of 928 patients had prostate cancer (18). Additionally, androgen signaling- therapies may provide a protective effect in infected cancer patients; these therapies are routinely used in the treatment of advanced prostate cancer, could restrict SARS-CoV-2 infection by down-regulating the expression of TMPRSS2 and stimulates an anti-SARS-CoV-2 immune response (15). Moreover, patients with prostate cancer treated with androgen-deprivation therapy (ADT) were found less likely to be infected by SARS-CoV-2 compared to prostate cancer patients not on ADT (19).

Further supporting the prioritization of cancer patients for care, is the finding that long term COVID-19 infection has been observed in cancer patients, with prolonged, viable, and infectious SARS-CoV-2 viral shedding (19–21). Studies by independent investigative teams identified viral shedding of SARS-CoV-2 beyond 70 days after infection in cancer patients on immunosuppressive therapy (i.e. chemotherapy, CAR-T or hematopoietic stem cell transplants) (21, 22). Patients with hematologic or metastatic cancer are more likely to have extended viral shedding than cancer patients with solid or localized tumors, with increased exhausted CD8+ T cells, and viral-induced lymphopenia (20).

To avoid SARS-CoV-2 infection, the surgical treatment strategy for cancer patients has been to delay non-urgent procedures, forego all but critical testing, and consider deferring treatment in low to intermediate risk cases until the risk of SARS-CoV-2 infection lessens. However, the long-term effect of deferred patient management on outcomes remains uncertain (23).

## Therapeutic Management

### Approved Repurposed Drugs

Throughout the pandemic many treatments have been developed or repurposed, aiming to treat or prevent COVID-19 (e.g., hydroxychloroquine, convalescent plasma, dexamethasone, remdesivir, immunomodulators, fluvoxamine) (24). Dexamethasone, a general anti-inflammatory agent, has shown efficacy in improving survival in hospitalized patients with severe COVID-19, and it’s use is recommended by the WHO and other medical societies (24). Remdesivir, a viral RNA-dependent RNA polymerase inhibitor, was originally tested against Ebola and Hepatitis C. It has been repurposed and was approved by the FDA for the treatment of COVID-19 requiring hospitalization (25). Remdesivir reduces hospitalization (26), however, other studies have shown mixed evidence (27). Baricitinib is a JAK inhibitor with immunomodulatory effects used in the management of rheumatoid arthritis. It received an emergency use authorization from the FDA for use in combination with remdesivier in patients with confirmed COVID-19 who require respiratory support (28). Tocilizumab, sarilumab and siltuximab are inhibitors of IL-6; a pathway associated with critical and fatal COVID-19. Two trials have indicated a mortality benefit for the use of tocalizumab in this setting and expert groups suggest the addition of tocilizumab to dexamethasone for hospitalized adults who have severe COVID-19 (29–32).

### Novel Therapeutics

Physicians first used convalescent plasma from flu patients to help others infected fight their infection in the 20^th^ century. In 2020, physicians tested convalescent plasma to combat COVID-19. Convalescent plasma treatment, rich with antibodies against SARS-CoV-2, has shown mixed efficacy. The FDA has permitted the use of convalescent plasma with a high concentration of antibodies. Also, the FDA has limited the use of convalescent plasma to hospitalized patients that are early in the course of COVID-19. SARS-CoV-2 antibody titers from previously infected patients have been stable for 5 months in patients that contracted the virus (33). Monoclonal antibodies have a long track record in modern medicine since their introduction to medicine in the 1970s. Currently, they are used to treat a variety of diseases including cancer.

In February 2021, the FDA announced emergency use authorization for the combination of the novel monoclonal antibodies bamlanivimab and etesevimab, which target overlapping epitopes in the SARS-CoV-2 spike protein, to treat mild to moderate COVID-19 (34). The monoclonal antibodies were isolated from two different patients that recovered from COVID-19 in North America (Bamlanivimab LY3819253 or LY-CoV555) and China (Estesevimab LY3832479 or LY-CoV-016). In a trial of 1,035 non-hospitalized adults with mild to moderate symptoms at high-risk for progression to severe disease the combination treatment significantly reduced the risk of hospitalization and death in the randomized, double-blind, placebo-controlled clinical trial (35).

## Vaccine Development

Currently, seven SARS-CoV-2 vaccines are approved for emergency use and are being administered. (i.e., Pfizer-BioNTech, Moderna, Gamaleya, Oxford-AstraZeneca, Sinopharm, Sinovac, Bharat Biotech). Advances in messenger RNA (mRNA) stability and efficiency in delivery have led to the development of several mRNA SARS-CoV-2 vaccines. mRNA vaccines are non-infectious, non-integrating, and devoid of infection risk or insertional mutagenesis. In December 2020, the FDA approved for emergency use authorization two lipid nanoparticle–encapsulated nucleoside-modified mRNA SARS-CoV-2 vaccines, BNT162b2 by Pfizer-BioNTech and mRNA-1273 by Moderna, with efficacy of 95 percent (36, 37). These vaccines are also approved for emergency use in the European Union and other countries. Both vaccines are a 2-dose series, a primer and booster dose, separated by 21 days (BNT162b2) or 28 days (mRNA-1273), use mRNA to induce an immune response specific to SARS-CoV-2 spike protein. The distribution and storage of the SARS-CoV-2 mRNA vaccines have created a logistic challenge because the vaccines must be stored at temperatures between −80 and −60°C (−112 and −76°F). The Ad26.COV2S (JNJ-78436725) is a recombinant, replication-incompetent adenovirus serotype 26 (Ad26) vector encoding the SARS-CoV-2 spike protein (38). The vaccine has been developed in partnership between pharmaceutical industry and academia, by Janssen Vaccines of Johnson & Johnson and Beth Israel Deaconess Medical Center. Ad26.COV2.S is a single-dose adenovirus vaccine approved for emergency use by the FDA in the USA in February 2021. A single dose of the vaccine provided 72 percent efficacy in clinical trial in USA, and 68 percent efficacy in Brazil and 64 percent efficacy in South Africa, in reducing moderate to severe COVID-19 disease 28 days after vaccination (38, 39). The vaccine can be refrigerated at 2 to 8°C. The AZD1222 Oxford-AstraZeneca vaccine, developed in collaboration between pharmaceutical industry (AstraZeneca) and academia (Oxford University) uses an adenovirus vector, ChAdOx1, to express the SARS-CoV-2 Spike protein (40). AZD1222 is a two-dose vaccine that has been approved for emergency use authorization in the United Kingdom and several countries in Asia and South America with 70.4 percent efficacy at reducing symptomatic COVID-19 infection 14 days after receiving the second dose (40). Additionally, other vaccines from Sinopharm, Sinovac, and Baharat Biotech vaccines are also inactivated. Gamaleya vaccine combines two adenoviruses, Ad5 and Ad26. AstraZeneca and Gamaleya are working in collaboration to test their vaccines as a combination vaccine. NVX-CoV2373 Novavax, a protein-based nanoparticle vaccine is an additional candidate currently being evaluated in clinical trials (41). The vaccine is a two-dose recombinant spike protein vaccine with proprietary adjuvant, MatrixM™ to enhance the response of the immune system to the vaccine. In the clinical trial, the vaccine had an 89 percent efficacy rate. Over 70 vaccine candidates are currently being investigated in clinical trials and 90 preclinical vaccine candidates are under investigation in pre-clinical models. By March 25, 2021, the WHO reported that a total of 432 million vaccine doses had been administered worldwide as of August 9^th^ 2021, this number was over 4 billion. Currently, approved SARS-CoV-2 vaccines are primarily administered to healthcare providers and the elderly. In the coming months, the vaccines will become more widely available to the general population including individuals with co-morbidities (e.g. cancer, diabetes, obesity and heart disease). The SARS-CoV-2 vaccines have not yet been tested in pediatric populations (e.g. school children required before returning to school), immune suppressed, or cancer cohorts. Currently, 17 million patients have a history of cancer in the United States. The available medical and scientific evidence are in support of the COVID-19 vaccine being considered high-priority for cancer patients, particularly patients with active cancer (42). Vaccine immunogenicity studies in patients with cancer conducted by Addeo et al. found that the SARS-CoV-2 mRNA vaccines produced high seroconversion with the second vaccine dose boosting antibody levels. Risk factors associated with non-responsiveness or low immunogenicity included hematologic malignancies, cytotoxic chemotherapy, and monoclonal antibody treatment (e.g. rituximab) (42, 43).

As global investigative efforts lead to further understanding of the design and composition of the SARS-CoV-2 vaccine, new approaches are being investigated to apply mRNA technology to fight cancer, as mRNA vaccines have the ability to stimulate the immune system to recognize and target cancer cells. Furthermore, the importance of vaccination is evident as more evidence on the long-term effects of COVID-19 become known. Some COVID-19 patients do not fully recover and continue to experience an array of symptoms that are debilitating. This syndrome encompasses symptoms such as chronic fatigue, fever, abnormal lung function (e.g. shortness of breath), and neurological conditions such as anxiety, depression and lack of concentration (44). While some viruses are known to be oncogenic (e.g. HBV, HCV, HIV, EBV and HPV) others are known to be oncolytic (e.g. adenovirus, reovirus and Coxsackievirus). The status of coronaviruses in this regard and the long-term effects of Long COVID are not well understood. While this form of COVID-19 continues to be defined it has been termed Post-Acute Sequelae of SARS-CoV-2 infection. Little is known about the long-term consequences of Long COVID-19 on cancer patients and outcomes. Physicians and scientists are conducting research into the prolonged health outcomes of SARS-CoV-2 infection on these patients known as “long haulers” (44, 45).

While data on the efficacy of these vaccines in GU Cancer patients is currently limited, our understanding will likely improve greatly as a result of concentrated global effort in the field of immuno-oncology. There is much translatable knowledge around immunosuppression and vaccination- for example the effects of anesthesia and surgery on immune response with vaccination during the perioperative period. Prior to the COVID-19 pandemic, the advisory committee on immunization practices at the Center for Disease Control recommended that vaccines be administered pre0operatiely or as soon as a patient’s condition stabilizes post-operatively (46, 47). Whilst definitive data in GU cancer patients is awaited, urologists and those who care for GU cancer patients are very familiar with and regularly rely upon intact immune responses when they administer Bacille Calmette-Guérin following transurethral resection of a bladder tumor. Current guidelines and position statements from the American Cancer Society, AACR, CDC and experts recommend that COVID-19 vaccination with authorized or approved vaccines should be made available to cancer patients (48–51).

## Clinical Diagnosis of GU Cancers

**Figure 2:**
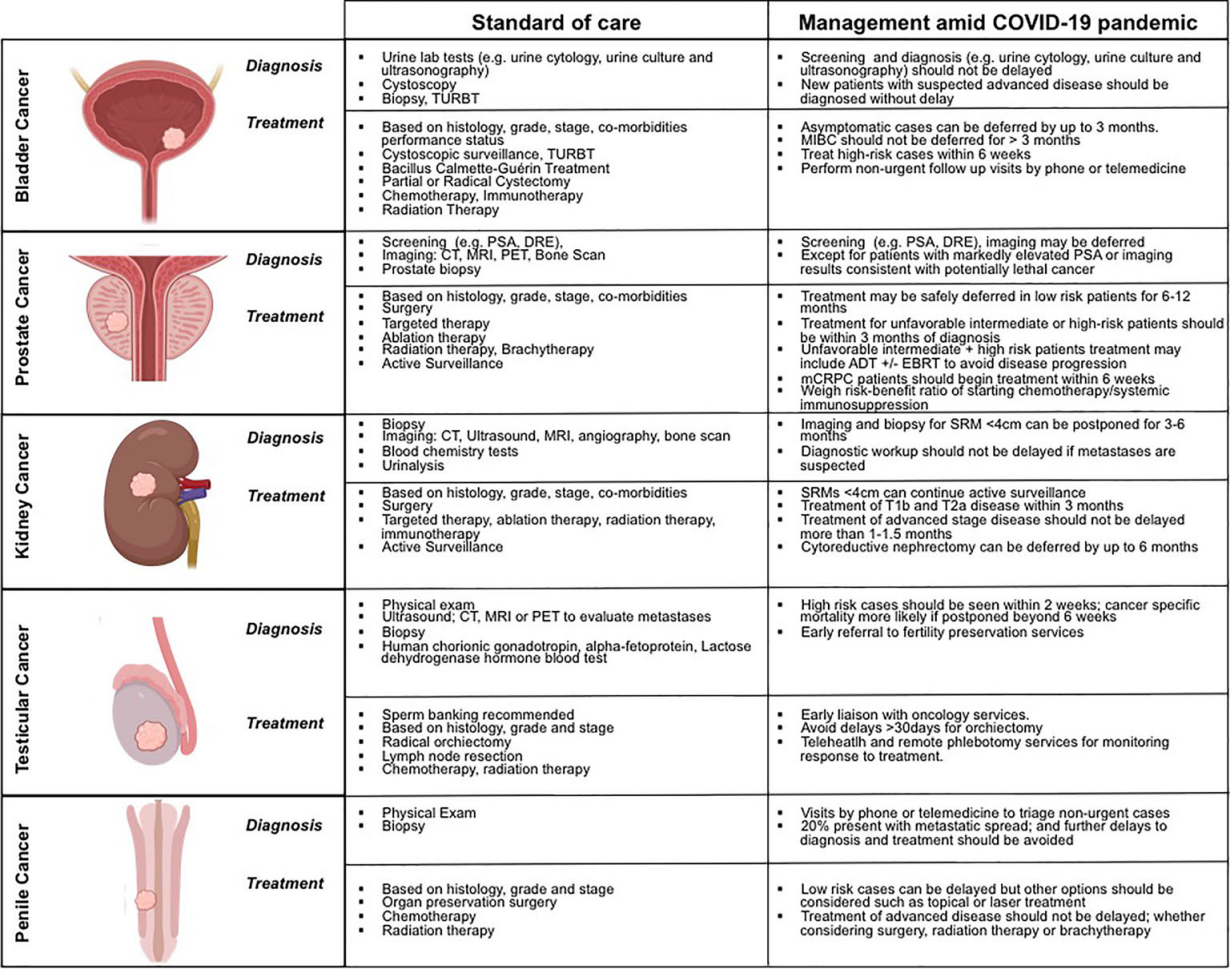
Contrasting the clinical diagnosis, management, and treatment of patientswith prostate, bladder, kidney, testicular, and penile cancers, before and during the COVID-19 pandemic.

The clinical diagnosis, management, and treatment of patients with prostate, bladder, kidney, testicular, and penile cancers, both the standard-of-care and during the COVID-19 pandemic are summarized on **Figure 2**.  These illustrate the great variability in the biology of these disparate cancers, including tumor growth kinetics, which affects the window in which diagnosis can be made, and the potential of a cure offered to these patients.

### Prostate Cancer

Prostate cancer is the most common malignancy in men worldwide (10). Approximately 60-75% of prostate cancers are located within the peripheral zone of the prostate (52, 53). Symptoms of prostate cancer manifest later in the disease process, and early detection largely relies on PSA (prostate specific antigen) and digital rectal exam screening, with a definitive diagnosis requiring a tissue biopsy. Additional risk stratification can be performed using MRI and blood tests such as the four-kallikrein panel (4Kscore^®^) (54). While the 15 year mortality rate is low in aggregate, beyond 15 years, a significant number of men, ultimately succumb to their disease (55). This low risk tumor profile represents half of all men diagnosed with prostate cancer (56). Prostate cancer progression to lethal disease remained relatively stable over the first 15 years, but increased threefold beyond 20 years follow-up (55, 57). The National Comprehensive Cancer Network (NCCN), European Association of Urology (EAU), and Canadian Urological Association (CUA) have released guidelines for prostate cancer screening during the COVID-19 pandemic (58–60). All of major international urology societies and organizations are in agreement that routine screening for asymptomatic individuals be deferred, except for patients with a very high PSA, or imaging results consistent with potentially lethal disease (58).

### Renal Cancer

The diagnosis of renal cell carcinoma (RCC) has shifted to that of masses identified incidentally with the widespread use of computed tomography (CT) scans for patients being worked-up for a variety of conditions or complaints. In a classic article from 1998, Jayson and Saunders noted through a series of 131 patients with RCC that 61% were diagnosed serendipitously during a workup for another cause. Only one patient had the classic triad of flank pain, hematuria, and an abdominal mass (61). The exponential increase in the use of CT scans has contributed to the rising incidence of RCC, yet the rate of RCC specific mortality remains stable (62). The increase in incidence has been particularly true for small renal masses (SRMs). With that, there has been a paradigm shift in the surgical treatment of all solid renal masses to that of active surveillance for SRMs (63). However, screening for renal masses was found not to be cost efficient and is not recommended (64, 65).

Considering the rate of metastasis for SRM is close to 1% (65), and 65% of patients have localized disease at the time of presentation (66), one can extrapolate that a delay in diagnosis of several months would not be clinically significant. Guidelines recommend that cross-sectional imaging or biopsy for SRMs (<4cm) could be delayed up to 6 months, whereas cT2 masses on ultrasound should undergo cross-sectional imaging within 3 months, while more urgent diagnostics are recommended for patients with suspected metastatic RCC (67).

### Urothelial Carcinoma

Urothelial carcinoma (UC) is one of the more aggressive cancers of the genitourinary tract. In fact, almost a third of all patients diagnosed will die within 5 years from their disease (68). The incidence is highest in patients aged 80-84 years, a group particularly vulnerable to COVID-19. Fortunately, 75% of patients have superficial disease at the time of presentation. The most significant prognostic factor is the stage, and particularly true for bladder cancer, grade of disease (69). Due to the lack of specificity in symptoms, some patients will face a delay in referral to a urologist and ultimately diagnosis (70). A delay in diagnosis has been found to signify an increased disease specific mortality (71). A clinical study identified nearly 30,000 patients in the SEER database to assess time from first visit for hematuria to diagnosis. This study found that a delay in bladder cancer diagnosis greater than 9 months had an increased risk of cancer specific mortality compared to those diagnosed within 3 months [HR 1.34]; the risk was still significantly increased after adjusting for grade and stage (71). Urology association guidelines suggest that patients with macroscopic hematuria should undergo a full diagnostic workup, including, but not limited to, CT program and cystoscopy with or without immediate tumor resection (72). An Italian study indicated that there has already been a significant decrease in cancer diagnoses during the COVID-19 pandemic, with a decrease in overall cancer diagnosis by 39% (73). On subtype analysis, prostate cancer had the most significant percent decrease in diagnosis (75%), followed by bladder cancer (66%), and colorectal cancer (62%), showing that the diagnosis of GU cancers may be disproportionately affected by the COVID-19 pandemic (73).

### Penile Cancer

While penile cancer is a rare malignancy and represents ~1% of all cancers affecting men, incidence increases from the age of 60 years and peaks in the eighth decade of life; an age group more vulnerable for adverse outcomes from COVID-19 (14). Penile cancer may be assigned a low priority in access to resources during COVID-19, because of the low incidence, but delays in care lead to significantly inferior patient outcomes (74). Most (~95%) penile tumors are squamous cell carcinomas with about half of these originating from non-keratinized epithelium of the glans or the inner layer of the prepuce. It is characterized by invasive growth and early metastatic spread to lymph nodes, where 20% of patients have metastases in the inguinal lymph nodes at presentation (75). Penile cancer is diagnosed with a biopsy (i.e., punch biopsy, fine needle biopsy, or elliptical excisions). Given that many penile cancers present late, and the risk for metastatic spread, pre-operative staging techniques may include inguinal ultrasonography, CT or MRI (76). The symptoms of penile cancer have led to patient delays in seeking treatment in the era prior to the COVID-19 pandemic, leading to an overall increase in the likelihood of disease progression (77). Delayed diagnosis of 6 months from a retrospective study noted that 47% of patients presented with locally advanced disease (78).

### Testicular Cancer

Testicular cancer is an uncommon malignancy, accounting for ~1-2% of all tumors in men. However it is the most common malignancy in young men (15-34 years); and is curable in most cases (79). While 95% of testicular tumors are of germ cell origin, Leydig cell tumors and lymphomas also occur. Typically the peak age of men with seminomatous germ cell tumors is 30-40 years, compared with 20-30 years for non-seminomatous germ cell tumors (NSGCT). Malignant Leydig cell tumors are observed more frequently in the elderly, and primary testicular lymphoma is the most common testicular malignancy in men older than 50 years of age. Testicular cancers usually present as a painless swelling of one testicle which are often incidentally noted. Approximately 10% of patients present with metastatic disease. Prompt diagnosis and treatment of testicular cancer provides the best opportunity for cure, as testicular tumors grow rapidly; with a tumor doubling time of 20-30 days (**Table 1**) (80).

**Table 1:** GU Tumor doubling times and their relationship to 5 year survival by disease extent.

GU Cancer	Primary Tumor doubling time (days)	5 Year survival for localized disease	5 year survival for regional	5 year survival for distant spread
Kidney	174-913	93%	70%	13%
Bladder	93-108	69% / 96%*	37%	6%
Prostate	900-2400	~100%	~100%	~30%
Penile	Days	80%	50%	9%
Testicular	10-30	99%	96%	73%

*For in situ disease.

Delays in testicular cancer diagnosis can affect the stage of disease at presentation and disease prognosis. Patients suspected of having testicular cancer are recommended to be seen within two weeks. Diagnostic evaluation includes serum tumor markers and scrotal ultrasound and is followed by staging CT imaging. Since treatment may cause infertility, patients should consider banking sperm prior to treatment (81, 82). The diagnostic, staging and banking required before treatment necessitates in person care; and while the presence of SARS-CoV-2 has been detected in semen of those recovering from COVID-19 (83) with impaired sperm quality was impaired in patients with moderate COVID-19 infection (84), these early findings would not require any change to the usual care, handling and storage precautions taken. It is of interest to note that an autopsy of six men in China with COVID-19 found that SARS-CoV-2 induced orchitis compared to healthy controls. In all of these patients, degenerated germ cells without changes to Sertoli cells were observed histologically (85). Other viruses (e.g. HPV, EBV, mumps) also have the ability to cause orchitis or testicular cancer (86). These findings are further supported by molecular studies using single-cell RNA-sequencing to evaluate SARS-CoV-2 tropism in human cells where ACE2 and TMPRSS2 were highly co-expressed in spermatogonial cells of the testes (8).

## Impact of COVID-19 on GU Cancer Treatment and Clinical Outcomes

### Prostate Cancer

Since the earliest emergence of demographic data from the COVID-19 pandemic, the gender disparity relating to infectivity and severity of disease course was evident; where males have a higher rate of hospitalization and mortality than females. Localized prostate cancer can be risk stratified into low, intermediate, and high risk. Several national organizations, such as the CUA and EAU, have provided treatment guidance for localized prostate cancer based on the risk category (58, 59). Very low risk and low risk disease, regardless of the COVID-19 pandemic, is generally safe to defer management, and nearly all published guidance is reflective of this (13, 58, 59, 87, 88). The European Association of Urology (EAU) recommends that for patients with low-risk disease on active surveillance, repeat PSA testing, DRE, and confirmatory biopsy can be deferred for 6 months, and patients seeking treatment (i.e. surgery or radiation therapy) should defer for 6-12 months (58).

Treatment of highly aggressive disease, such as unfavorable intermediate and high risk cancer that will lead to lethal disease, should be prioritized. Updated guidelines recommend treatment of unfavorable intermediate risk and high-risk disease within 3 months of diagnosis (87). However, treatment in these instances may include ADT alone to avoid progression of disease, or ADT with external beam radiation (87). It is important to note that one retrospective study of 2,500 patients found that a treatment delay in intermediate and high risk patients greater than 2.5 months conferred a higher risk of biochemical recurrence (89). In contrast, another study found no difference in adverse outcomes for delayed surgery up to 6 months with 10 year follow up (90). While in person visits and discussions may be limited, there is less of a limitation on multi-disciplinary discussion and formulation of individualized treatment plans to balance the risks of treatment delay of localized cancer and benefits of reducing unnecessary exposures to a vulnerable patient population (13). For patients diagnosed with metastatic castration-resistant prostate cancer (mCRPC), treatment is a high priority which should begin within 6 weeks (58). While the majority of approved agents have required in person injection or implantation or patient education to self-administer, in December 2020, relugolix (Orgovyx), the first oral gonadotropin-releasing hormone (GnRH) receptor antagonist, was approved by the FDA. Relugolix is an oral alternative to ADT administered by injection or subcutaneous implant and provides another treatment option for patients (91).

Society guidelines further recommend that other AR-targeted therapy, such as second generation antiandrogens, enzalutamide or apalutamide, should be continued. There are concerns that the use of first-line taxane chemotherapy with docetaxel is likely of greater risk than benefit, as many patients will experience neutropenia as a side effect, thus making them more susceptible to morbidity and mortality from COVID-19 (92). The impact of hormone therapy (anti-androgens) on prevention or reduction of COVID-19 symptoms is under investigation (4), with the rationale that the Transmembrane Serine Protease 2 (*TMPRSS2*), a SARS-CoV-2 entry factor, is an androgen-regulated gene (93). The upregulation of TMPRSS2 by androgen is mediated by the androgen receptor (AR). TMPRSS2 is highly expressed in prostate epithelial cells and is one of the most dysregulated genes in prostate cancer (93). Several clinical trials are currently ongoing nationwide to test the androgen/AR signaling inhibitors (second generation antiandrogens such as enzalutamide and abiraterone) for therapeutic targeting of SARS-CoV-2 internalization and treatment of patients with COVID-19 (4).

### Renal Cancer

While RCC and COVID-19 share biological mechanisms, and treatment of one may have beneficial effects on the other, the treatment of kidney cancer is determined by the clinical stage as well as the growth kinetics of individual tumors (94). Small renal masses (<4cm) are often safe to undergo active surveillance. The median growth rate of SRMs is 1.9mm/year. The effect of delayed intervention for SRMS was previously studied and with follow up to 5 years, 47% of patients underwent definitive surgery, ~1% developed metastatic disease, and delayed treatment was deemed to not have an impact on overall survival (95). The AUA guidelines during non-pandemic times encourage active surveillance with deferred treatment for SRMs (96), with similar treatment guidance issued by the EAU during the COVID-19 pandemic (67).

Data regarding treatment deferrals for patients with T1b, T2, and T3 disease is less robust. One retrospective study of 319 patients found no difference in cancer specific outcomes or mortality for patients with T2 disease who underwent nephrectomy within 1 month of diagnosis compared to those who waited 1 to 3 months (97). Another study found that growth kinetics of T1b tumors were similar to SRMs, and can be managed initially with a trial of surveillance in select patients, to assess growth rate without significant effects on tumor resectability or cancer specific outcomes (97, 98). The EUA recommends treating patients with T1b and T2a disease within 3 months during the COVID-19 pandemic (67). There is a paucity of data on delayed treatment for T3 disease. One patient, with a level I-II IVC thrombus progressed to a level III thrombus after foregoing treatment for 30 days (99). The available evidence suggests that treatment of clinically advanced disease should not be delayed: patients with metastatic disease should also not forego life-prolonging chemotherapy or immunotherapy, and individual groups have proposed management algorithms for advanced RCC patients during COVID-19 (100). Cytoreductive nephrectomy can be deferred by up to 6 months (67). Guidance from experts recommend that first line treatment for intermediate and high risk disease with immunotherapy should be commenced (92). However, asymptomatic patients with minimal disease and favorable or intermediate risk disease can be considered for active surveillance, as the median progression free survival was 9.4 months and overall survival 44.5 months (67, 101). (**Figure 2**). The National Comprehensive Cancer Network also provides a toolkit to assist transitioning inpatient chemotherapy regiments to the outpatient setting (102).

### Urothelial Carcinoma

Bladder cancer is often an aggressive disease, yet the majority are low stage (Ta/Tis) at the time of diagnosis (103). For patients with low-grade, non-muscle invasive cancer(NMIBC), there is a very low rate of progression, with a bladder cancer specific mortality of approximately 1-2% (104). Thus it is potentially safe to defer treatment/surveillance of patients with low-grade disease NMIBC, as international societies guide to deferring endoscopic tumor resection and intra-vesical therapy up to 6 months (73). High-grade NMIBC is considered an entirely different disease process with a much more aggressive course than low-grade disease. Progression to muscle invasion occurs in 15-40% of patients and disease specific mortality rises to 10-20% (105). Outside of endoscopic resection, a key component to the management of these patients is intravesical immunotherapy, particularly Bacillus Calmette-Guerin (BCG). The safety of intra-vesical BCG during the COVID-19 pandemic has not been thoroughly interrogated. However, as the mechanism of action potentially involves the immune response, it has been postulated that BCG may be protective against COVID-19, though these results have not been specific to intravesical therapy (105). Given the aggressive nature of high-grade NMIBC and potentially varied presentations and stages of treatment, the EAU recommends resection deferral of asymptomatic patients by 3 months with BCG within 6 weeks. Patients who have incomplete resections should undergo re-resection within 6 weeks. Patients who failed BCG therapy should be offered radical cystectomy within 3 months to avoid negatively impacting disease specific survival (73).

Muscle-invasive bladder cancer (MIBC) is an aggressive disease for which treatment should be scheduled within 3 months to prevent an increase in disease specific mortality (106). The metric for treatment delay is not standardized, with some studies reporting time from transurethral resection of bladder tumor (TURBT) to radical cystectomy, others from diagnosis to radical cystectomy, and some from neo-adjuvant chemotherapy to radical cystectomy (49). Exemplifying the impact of timely treatment, Kulkarni et al. found that risk of death from bladder cancer begins to increase 40 days after resection, but prior to definitive treatment (107). Patients with greater than 10 weeks between neo-adjuvant chemotherapy and radical cystectomy had a significantly lower cancer-specific and overall survival (108) (**Figure 2**). The majority of studies identified a significant risk in delaying definitive treatment with cystectomy greater than 3 months, and accordingly the recommendation is for cystectomy within 3 months for MIBC (109) with frontline chemotherapy for metastatic disease not to be delayed (91), despite some data suggesting that among patients treated with systemic cytotoxic chemotherapy within three months of COVID-19 diagnosis, including combinations of platinum drugs, are associated with an elevated risk of mortality (110). While some guidelines have advised avoiding the use of immuno-oncology agents during COVID-19 because of the potential for immunosuppression and increased risk of acute respiratory distress syndrome that develops during active infection, there is conflicting evidence at this time as to whether such therapies affects the severity of COVID-19 (92, 111). A meta-analysis of active cancer treatment on severity of COVID-19, identified that chemotherapy within the last 30 days but not immunotherapy within the past 30 days, had a higher risk of death from COVID-19, when controlling for potentially confounding factors (112).

### Penile Cancer

Typically patients with penile cancer present late in the course of the disease, and delaying penile cancer treatment can lead to disease progression of the primary tumor such that organ preserving surgery, may no longer be a treatment option (113). In a recent retrospective analysis, early surgical intervention for penile cancer demonstrated 5 year disease specific survival of 61.4% vs 39.5% in those with delayed dissection (114). Despite potential immunomodulatory effects, consensus guidelines recommend that those patients with advanced or metastatic disease should be considered for neo-adjuvant or adjuvant chemotherapy with or without radiation therapy (77) (**Figure 2**). For penile cancers with intermediate risk of progression, surgical treatment may be delayed up to three months, but radiation therapy and brachytherapy should be considered as effective options. Follow-up visits can where appropriate, be facilitated by telemedicine tools. Prognosis is determined by several characteristics, the presence of lymph nodes being the most important. In such cases, treatment should not be delayed. Histological diagnosis with local staging is necessary before offering any therapeutic option. In the case of superficial non-invasive disease, topical treatment is effective in the absence of lymph node involvement. In selected patients, radiotherapy is an organ-preserving approach with good results (**Figure 2**). Non-deferrable surgical treatment must be performed as an outpatient procedure when possible.

### Testicular Cancer

Early treatment of patients with testicular cancer includes radical inguinal orchiectomy with or without retroperitoneal lymph node dissection. While almost all seminomas are curable with surgery, and are radiosensitive and chemo-sensitive, NSGCT are less sensitive to radiation and when metastatic often require both chemotherapy and surgery. There are also differences in the cure rates for testicular tumors in the elderly compared with younger patients. In the pre-cisplatin chemotherapy era, the impact of prompt (<30days) vs. delayed (>30days) orchiectomy for NSGCT was shown to lead to significant progression of disease. Those in the prompt orchiectomy group had significantly more stage 1 tumors, while delayed had significantly more stage 3 tumors (115). However with the advent of effective chemotherapy regimens, there is no difference in survival rates between early and delayed surgery (13, 116).

### Novel Diagnostics

The COVID-19 pandemic presents a unique opportunity for innovative diagnostics. Limiting viral exposure by utilizing novel blood or urine assays (e.g. liquid biopsy), radiomics, and machine learning could help identify those at highest risk for cancer progression (117–119). The basis of a liquid biopsy involves a solitary underlying mechanism regardless of tumor type. Blood samples can contain material from different tissues, including tumors. Of importance, cell-free DNA (cfDNA), RNA [i.e. primarily microRNA (miRNA)], exosomes, and circulating tumor cells (CTCs) have enabled “non-invasive” evaluation of a patient tumor status (120). These molecular markers can also be shed into the urinary tract and identified in urine. Further, radiomics has the ability to utilize a large volume of data to identify imaging characteristics that may not only detect the presence of malignant or benign disease, but also identify clinically significant disease processes, as in the case of prostate cancer (121).

There are four commercially available urinary biomarkers for prostate cancer: Progensa PCA3, SelectMDx, Michigan Prostate Score (MiPS), and ExoDx Prostate (IntelliScore). These urinary biomarkers all share the common benefit of a negative predictive value (NPV), ranging from 90% for PCA3, and 98% for SelectMDx (54). PCA3 outperformed PSA alone in predicting a positive prostate biopsy, but has limited value in predicting clinically significant versus insignificant disease (122). Blood-based biomarkers for prostate cancer have been limited in terms of diagnostics, and studies thus far have been in the setting of identifying response to therapy in metastasis. Thus, the mainstay of blood biomarkers are PSA and its derivatives, such as prostate health index (PHI), or the four-kallikrein panel (4KScore), which utilizes total PSA, free PSA, intact PSA, and human kallikrein-related peptidase (122). In December 2020, a new PET imaging method, the Gallium 68 PSMA-11 (PSMA PET), was approved by the FDA for detection of prostate cancer metastases. The PSMA PET scan is indicated for patients suspected to have metastatic cancer. Compared to CT, bone scans, and MRI, PSMA PET is more sensitive for use for initial management decisions to assess the presence of metastases (123).

Many candidate urinary biomarkers for RCC have low sensitivity and specificity, and have been unable to differentiate between benign renal masses from malignant renal tumors. Urine biomarkers and liquid biopsy with circulating tumor DNA are also areas of active investigation. Multiple FDA-approved tests are available for bladder cancer using DNA, mRNA, protein, or sediment in the urine. These tests can be used for diagnosis or follow-up. One example is UroVysion™ from Abbott Laboratories. UroVysion™ is a fluorescent *in situ* hybridization (FISH) probe assay that detects bladder cancer cells in the urine (124). This assay uses fluorescent labeled DNA probes to assess chromosomal alterations such as aneuploidy of chromosomes 3, 7, and 17, and loss of the 9p21 locus. The sensitivity and specificity are 63–72% and 85–87%, respectively. In October 2020, the FDA approved Liquid CDx from FoundationOne, a comprehensive genomic profiling assay for solid tumors (125). This pan-cancer liquid biopsy test uses serum from blood to analyze over 300 genes from cell-free DNA to assist in clinical decision making. Liquid CDx reproducibly detects with the genomic alterations, microsatellite instability, and mutational burden. Liquid biopsies are non-invasive, and can be used as a companion diagnostic test to inform personalized treatment decisions (126). Pathological classifications of renal, prostate, and urinary tumors can be subtyped by molecular immunostaining profiles of the tumor microenvironment to provide additional diagnostic information (127).

## The Emergence and Impact of SARS-Cov-2 Variants

Coincident to worldwide COVID-19 vaccination deployment, several SARS-CoV-2 variants have emerged. The development of these variants have been expected as RNA viruses typically have higher mutation rates compared to DNA viruses triggering a remarkable momentum in research on global scale to effectively track the spread of SARS-CoV-2 and variants. As investigators are aggressively interrogating the contagious nature, virulence, transmission, and existing vaccine efficacy against the variants, the primary focus of genomic surveillance of the SARS-CoV-2 variants has been on the acquired mutations in the spike protein. Indeed, there is significant interest in understanding whether spike protein mutations will provide the virus with the ability to escape host antibodies and potentially lessen the efficacy of the SARS-CoV-2 vaccines (128). Here, we briefly summarize some of the variants that have been identified worldwide which could prolong the impact of this pandemic on the provision of GU cancer care to patients.

The WHO and other biomedical research groups are currently developing a nomenclature for the various SARS-CoV-2 variants (129, 130). B.1.1.7, VOC202012/01 “UK variant” (UK, December 2020) has spread to 62 countries including the USA. The variant contains 17 lineage-defining mutations including N501Y, P681H, HV 69–70 deletion of which 8 of the mutations are located in the spike protein including ΔH69/ΔV70 which is associated with immune escape in immunocompromised patients, and enhanced viral infectivity (131). The COVID-19 UK variant accounts for about 28% of SARS-CoV-2 cases in England (131), and has a higher transmission rate (131). The B.1.351, 20C/501Y.V2 variant (South Africa, December 2020) is currently present in Africa, Europe, Asia, Australia, and the USA. This variant contains 21 mutations including the N501Y, E484K, and K417N mutations located on the spike protein. P.1, 20J/501Y.V3 (Japan and Brazil, January 2021) has spread to the Faroe Islands, South Korea, and the USA. The 17 amino acid changes identified include the N501Y mutation, which is located on the viral spike protein and observed in other variants. Other changes include E484K, and K417N on the spike protein, and ORF1b deletion, which is located on the outside of the spike protein. Cases of reinfection with this variant have been reported in Brazil, however, the potential for immune evasion of P.1, 20J/501Y.V3 remains unknown. COH.20G/501Y (USA, December 2020) was originally identified in Ohio, USA and has spread to other states within the USA (132). Another USA strain, B.1.526 (USA, November 2020), was identified in New York City, as two distinct variant forms. One variant harbors the E484K spike protein mutation, while the other has the S477N mutation. The E484K mutation has also been identified in the B.1.351 variant and is associated with immune escape. Vaccines developed against the original wild-type virus have been found to be less effective against B.1.351 (39). Variant Fin-796H also harbors the E484K mutation. In February 2021, the B.1.526 variant accounted for about 27% of infections in New York, NY, USA. Variant CAL.20C (November 2020, USA) was identified in California, USA in late 2020 (133) and by February 2021, the variant accounted for about 50% of samples tested in Los Angeles, CA, USA. Currently, of these variants, B.1.1.7, B.1.351, and P.1 are being closely monitored because of their suspected high transmissible risk, leading to severe illness, and ability to elude the immune response after infection or vaccination (5). The coronavirus variants, B.1.617.1, B.1.617.2 and B.1.617.3, first identified in India, have generated a massive surge in cases and deaths in India, shown increased transmissibility, co-infection with mucormycosis (black fungus) in a sub-set of patients, and has spread worldwide (134). A study has shown that Pfrizer-BioNTech or AstraZeneca-University of Oxford vaccines protect against these variants (135).

## Health Disparities in Outcomes in SARS-CoV-2 Infected Patients With GU Cancers

Several factors including patient race, health literacy, and low socioeconomic status and its correlates (e.g., limited access to health care, lack of medical insurance, Zip Code) have been repeatedly linked to differences in GU cancer incidence, morbidity, and mortality (136). Published data on SARS-CoV-2 characterizes the resulting COVID-19 disease as one exploiting existing inequities, and thus, may contribute to increased health disparities in chronic disease patient populations including cancer (137–140). Increasing evidence from SARS-CoV-2 research showed that the pandemic will exacerbate existing disparities as Black/African Americans and Hispanic/Latinx are more likely to be diagnosed and experience COVID-19-related morbidities and mortality, especially those living in poor and crowded housing conditions, having pre-existing health comorbidities, low/limited incomes, or “essential” occupations (138). Moreover rapidly increasing inequities in disease burden emerge among other minorities including Native American/American Indians (NA/AI) (138). Among cancer patients, the COVID-19 pandemic has created different challenges across the continuum of cancer care, with potentially significant clinical consequences of delays in cancer screening, diagnosis, and treatment (141, 142). A recent EPIC Health Research Network study (143) using data from 190 hospitals spanning 23 states showed a 85% decrease in the number of screening tests for cervical, breast, and colon cancer conducted after the first COVID-19 case was reported in the US on January 20, 2020. Another study showed that 79% of patients with cancer reported delays in their active cancer care as a result of COVID-19 (144).

The US National Cancer Institute (NCI) estimates there will be approximately 10,000 additional deaths from breast cancer and colorectal cancer over the next decade due to the impact of COVID-19 on cancer care (141). Resulting delays in cancer screening and treatment for these two types of cancer raise concerns about similar long-term impact on GU cancers, specifically bladder cancer given the high recurrence and tumor progression rates (141). Additionally, due to COVD-19, major previous barriers to telehealth use including poor financial reimbursement and low provider willingness were eliminated essentially overnight, thus massively speeding up adoption (145). However, a study examining racial differences in the utility of telehealth after COVID-19 in a New York City health system showed that, compared to white cancer patients, Black/African American and Hispanic cancer patients were more likely to use emergency room (ER) and office visits as compared to telehealth visits. Compared to any age group, older cancer patients (>65 years) were more likely to use either ER or office visits than telehealth (145). Further increasing COVID-19 related cancer disparities, many patients, the majority of whom are ethnic minorities, lost their income due to unemployment, thus, leading to loss of health insurance and, consequently, access to cancer care (136). Addressing cancer disparities related to COVID-19 will improve our understanding of factors that contribute to COVID-19 related cancer disparity and coordinate efforts to address the added burden of the pandemic on cancer patient outcomes.

## Conclusions

The global pandemic has presented the opportunity for greater adoption of novel methods to diagnose, treat, and interact with patients through the greater adoption of technologies, both digital and scientific. Reluctance in engaging with the healthcare system during the SARS-CoV-2 pandemic has led to delays in reporting symptoms, cancer diagnosis, and consequently the progression of undiagnosed GU cancers (**Figure 1**). An extended follow-up time is necessary to better understand the long-term impact of COVID-19 on clinical cancer outcomes (i.e. progression, survival) of patients with GU cancers including but not limited to the ability to accurately diagnose tumors, effectively receive or continue cancer treatments, and minimize tumor progression or recurrence while reducing SARS-CoV-2 infection (**Figure 2**). We recognize that COVID-19 has presented a unique, generational moment for GU cancer physicians and research investigators. Moving forward, the global scientific community faces both opportunities and challenges to prospectively address critical questions at (a) the molecular level to identify overlapping mechanisms driving the two diseases and shared actionable targets; and (b) the clinical setting providing a powerful platform for evidence-based disease surveillance and treatment monitoring.

## Author Contributions

Conception: SI, DL, VP, MG, PW, WO, C-KT, AT, and NK. Research material: SI, DL, NM, AK, and BE. Writing: SI, DL, NM, RM, JS, MG, and NK. Reading: SI, DL, VP, RM, JS, KB, WO, NM, CC-C, AT, MG, and NK. Supervision: NK. All authors contributed to the article and approved the submitted version.

## Conflict of Interest

C-KT has financial relationships in consulting with Clovis, Pfizer, and Eisai. WO has financial relationships in consulting with Astellas, Astra Zeneca, Bayer, Janssen, Sanofi, Sema4, and TeneoBio. AT has financial relationships in consulting with Intuitive Surgical, Promaxo, Roivant, Siemens, and Kite Pharma. He serves as an advisor for and owns equity in the form of stock certificates in Promaxo. MG has financial relationships in consulting with BioMotiv, Janssen, Dendreon, Merck, GlaxoSmithKline, Lilly, Astellas Pharma, Genentech, Bristol‐Meyers Squibb, Novartis, Pfizer, EMD Serono, AstraZeneca, Seattle Genetics, Incyte, Aileron Therapeutics, Dracen, Inovio Pharmaceuticals, NuMab, and Dragonfly Therapeutics. Matthew Galsky has received research funding from Janssen Oncology, Dendreon, Novartis, Bristol‐Myers Squibb, Merck, AstraZeneca, and Genentech/Roche. MG also has financial ownership interest in Rappta Therapeutics.

The remaining authors declare that the research was conducted in the absence of any commercial or financial relationships that could be construed as a potential conflict of interest.

## Publisher’s Note

All claims expressed in this article are solely those of the authors and do not necessarily represent those of their affiliated organizations, or those of the publisher, the editors and the reviewers. Any product that may be evaluated in this article, or claim that may be made by its manufacturer, is not guaranteed or endorsed by the publisher.

## Abbreviations

ACE2, angiotensin-converting enzyme 2; Ad26, adenovirus serotype 26; ADT, Androgen-deprivation therapy; CAR-T, chimeric antigen receptor T-cell therapy; CDC, Center for Disease Control and Prevention; COVID-19, Coronavirus Disease 2019; CT, computed tomography; CUA, Canadian Urological Association; DRE, Digital Rectal Exam; EAU, European Association of Urology; ER, Emergency Room; EUA, Emergency Use Authorization; FDA, United States Food and Drug Administration; FISH, Fluorescent In Situ Hybridization; JAK, Janus Kinase; MIBC, Muscle-invasive bladder cancer; mRNA, messenger RNA; NCI, National Cancer Institute; NCNN, National Comprehensive Cancer Network; PSA, Prostate Specific Antigen; PSMA, Prostate Specific Membrane Antigen; RCC, Renal Cell Carcinoma; SARS-CoV-2, severe acute respiratory syndrome coronavirus 2; SEER, Surveillance, Epidemiology, and End Results Program; TMPRSS2, transmembrane protease serine 2; USPTSF, United States Preventive Services Task Force; WHO, World Health Organization.
